# Histopathological Features and Antiviral Efficacy in Patients Over 30 Years Old with Chronic Hepatitis B in the Indeterminate Phase

**DOI:** 10.5152/tjg.2025.25138

**Published:** 2025-10-03

**Authors:** Ke-Ying Ou, Yue-Yang Ma, Chuan-Su Yuan, Yu-Jia Lu, Bin Liu, Jia-nan Cui, Wei-hao Zhao, Yong-Feng Yang, Qing-Fang Xiong

**Affiliations:** 1 Department of Infectious Disease and Liver Disease, the Second Hospital of Nanjing, Affiliated to Nanjing University of Chinese Medicine, Jiangsu, China; 2 The Clinical Infectious Disease Center of Nanjing, Jiangsu, China; 3 Clinical Research Center, The Second Hospital of Nanjing, Nanjing University of Chinese Medicine, Jiangsu, China

**Keywords:** Antiviral agents, chronic, fibrosis, hepatitis B, indeterminate phase, inflammation

## Abstract

**Background/Aims:**

There are still many controversies about whether antiviral therapy should be administered to patients with chronic hepatitis B (CHB) in the indeterminate phase. Thus, this study aimed to investigate the histopathological features and antiviral efficacy of HBeAg-negative CHB patients aged over 30 in the indeterminate phase.

**Materials and Methods:**

The clinical, laboratory, and histopathological characteristics of 666 CHB patients were assessed through a retrospective study. To identify factors associated with significant liver inflammation and fibrosis, inter-group differential analysis and binary logistic regression were conducted. Receiver operating characteristic curve analysis was used to determine the area under the curve and optimal cut-off values for relevant indicators. The antiviral efficacy was analyzed in 62 patients who received antiviral treatment by inter-group differential analysis.

**Results:**

A total of 70 patients were enrolled in the study. The median age was 40.5 years and 38 patients (54.28%) were male. Significant liver inflammation and significant liver fibrosis represented 30% and 55.7% of the patient cohort, respectively. Multivariate logistic regression analysis revealed that red blood cell count (RBC) and aspartate aminotransferase (AST) were independent predictors of significant liver inflammation (odds ratio [OR] (95%CI): 0.34 (0.13,0.90), *P* = .03; OR (95%CI): 1.19 (1.05,1.34), *P* = .006). Aspartate aminotransferase was also an independent predictor of significant liver fibrosis (OR (95%CI): 1.24 (1.06,1.47), *P* = .01). The negative conversion rate of hepatitis B virus (HBV) DNA was above 80% from 24 weeks after antiviral treatment, and low-level viremia (LLV) accounted for 6.5% (4/62) at 48 weeks after antiviral treatment.

**Conclusion:**

Among HBeAg-negative CHB patients aged over 30 in the indeterminate phase, over half had significant liver inflammation or fibrosis. In addition, RBC was a protective factor for significant liver inflammation, and AST was a risk factor for significant liver inflammation and fibrosis in such patients. Notably, antiviral treatment was effective. However, long-term monitoring of HBV DNA and the occurrence of LLV and drug resistance should be conducted during treatment.

**Main points**

- Among the HBeAg-negative chronic hepatitis B (CHB) patients aged over 30 in the indeterminate phase, more than half of them had significant liver histological changes.
- In HBeAg-negative CHB patients aged over 30 in the indeterminate phase, red blood cell count is a protective factor for significant liver inflammation, and aspartate aminotransferase is a risk factor for significant liver inflammation and fibrosis.
- Antiviral treatment is effective for CHB patients in the indeterminate phase, but attention should be paid to the occurrence of low-level viremia and drug resistance.

## Introduction

In 2016, the World Health Organization (WHO) established a strategic objective to eliminate viral hepatitis as a global public health concern by the year 2030, aiming to reduce new infections by 90%, decrease mortality by 65%, and enhance diagnostic and treatment rates to 90% and 80%, respectively.^1^ In alignment with this goal, the 2022 revision of the Chinese “Guidelines for the Prevention and Treatment of Chronic Hepatitis B (CHB)” proposed expanding the indications for antiviral treatment.^2^ It advised initiating antiviral treatment for CHB patients over 30 years old with persistently normal alanine aminotransferase (ALT) and suggested active treatment for patients in the indeterminate phase who have been followed up for more than 1 year. The guideline highlights the challenges in categorizing certain treatment-naive patients with 1 year of follow-up into distinct phases—namely, the immune tolerance phase, immune clearance phase, immune control phase, and reactivation phase—based on their HBV DNA levels, ALT levels, and liver histology. However, some patients do not fall into the above 4 stages and are classified as “indeterminate phase.”^2^ Research indicates that between 27.8% and 59.5% of patients with chronic HBV infection are still in the indeterminate phase, with nearly 50% of them exhibiting significant liver fibrosis or inflammation on liver biopsy.^3^^-^^8^ However, the efficacy of antiviral treatment for patients in the indeterminate phase remains inadequately understood. Zhao et al^9^ and Huang et al^10^ reported that long-term antiviral treatment can significantly reduce the incidence of cirrhosis and hepatocellular carcinoma (HCC) by reducing HBV DNA concentrations. However, some patients are presenting with low-level viremia (LLV, defined as detectable HBV DNA after 48 weeks of antiviral treatment, but < 2000 IU/mL^2^) with the widespread use of antiviral medications, even progressing to liver cancer.^11^

This study aims to find a simple, non-invasive surrogate marker and evaluate the safety and efficacy of antiviral therapy in HBeAg-negative CHB patients aged over 30 in the indeterminate phase, so as to provide evidence-based medical evidence.

## Materials and Methods

### Patients

**Figure 1.**
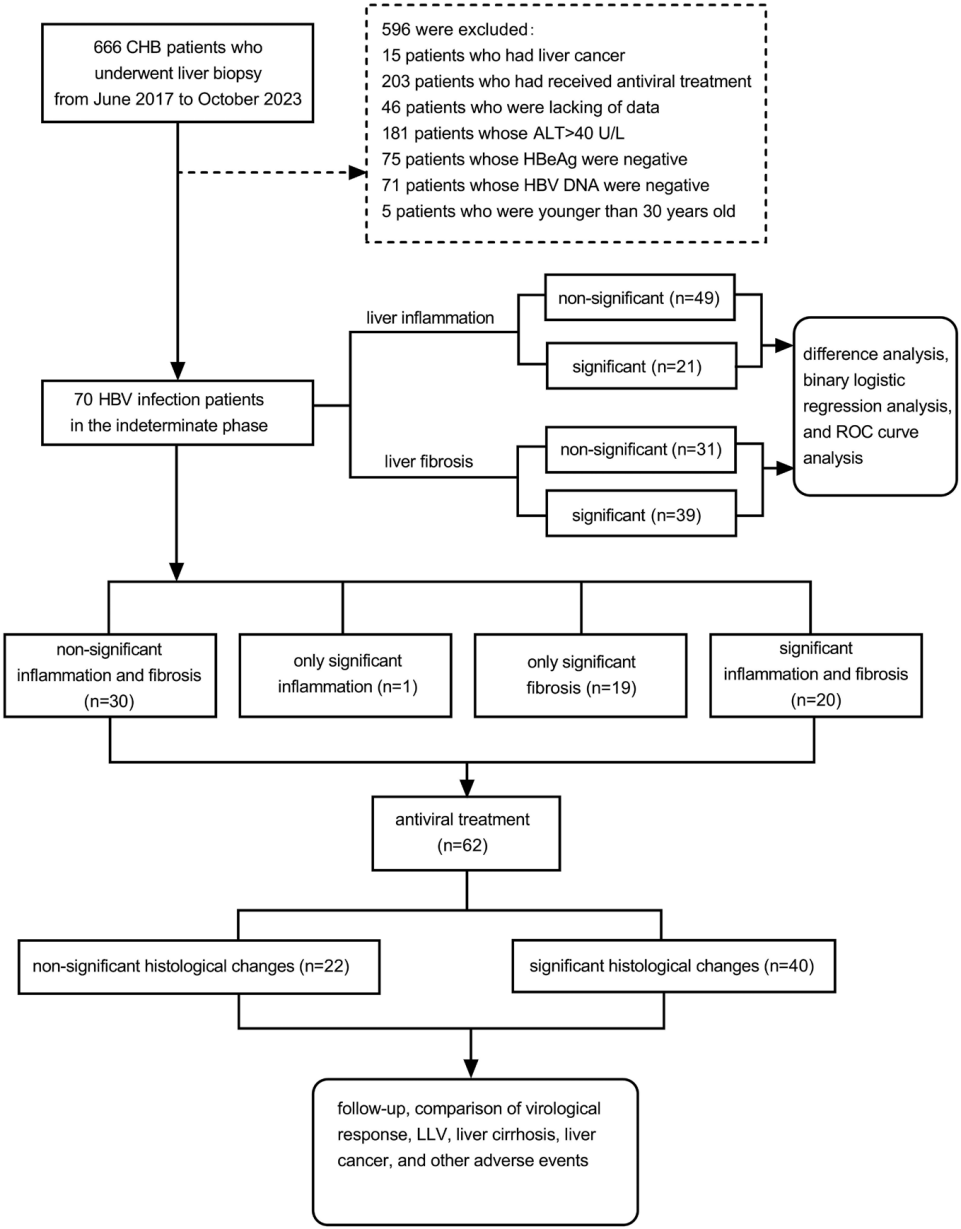
Study design flowchart. Abbreviations: ALT, alanine aminotransferase; LLV, low-level viremia; ROC, receiver operating characteristic.

Between June 2017 and October 2023, 666 chronic hepatitis B patients with liver biopsies performed at the Second Hospital of Nanjing were enrolled in a retrospective analysis. The following were excluded: 15 patients with liver cancer, 203 patients with antiviral treatment or antiviral drug resistance, 46 patients with incomplete data, 181 patients with ALT > 40 U/L, 75 patients with positive HBeAg, 71 patients with undetectable HBV DNA, and 5 patients under 30 years old. Ultimately, 70 patients were included, as shown in Figure 1. All patients provided informed consent. The study protocol was approved by the Medical Ethics Committee of the Second Hospital of Nanjing (2023-LS-ky-039, date: November 16, 2023). Written informed consent was obtained.

### Enrollment Criteria

#### Inclusion Criteria:

The HBeAg-negative CHB patients aged over 30 in the indeterminate phase were included. According to the 2022 revision of the Chinese “Guidelines for the Prevention and Treatment of CHB,”^2^ the specific requirements are as follows: (1) CHB patients who had not used any antiviral drugs within 1 year. (2) ALT < 40 U/L. (3) HBeAg < 1.00 COI. (4) HBV DNA > 500 IU/mL. (5) Age > 30 years.

#### Exclusion Criteria:

(1) Individuals with confirmed diagnoses of liver cancer or other viral hepatitis diseases. (2) Individuals who are currently undergoing antiviral treatment or who received it within 1 year prior to liver biopsy. (3) Individuals lacking information regarding antiviral treatment status, liver function tests, HBeAg levels, HBV DNA levels, and other relevant examination data.

### Laboratory Testing Indicators

The BC-3000 blood cell analyzer (Mindray, Shenzhen, China) was used for routine blood examination; the AU2700 automatic biochemical analyzer (OLYMPUS, Japan) was used to measure biochemical indicators; the automatic chemiluminescence analyzer (Abbott, USA) was used to detect Hepatitis B Serological Markers; the Step One Plus Polymerase Chain Reaction (PCR) fluorescence analyzer (lower limit of detection at either 500 IU/mL or 20 IU/mL; Roche Diagnostic System Inc., Branchburg, USA) was used to detect HBV DNA.

### Liver Biopsy and Pathological Diagnosis

A liver biopsy was performed with a 16G needle and an automatic adjustable biopsy gun (Bard, USA) under ultrasound guidance. Each specimen was required to have a minimum length of 2 cm and contain 11 portal tracts. Evaluation of all tissue samples was performed independently by 2 pathologists using an optical microscope. Liver inflammation (G) and fibrosis (S) were graded according to the Metavir scoring system,^12^ with significant inflammation specified as G ≥ 2 and significant fibrosis defined as S ≥ 2. Patients with significant inflammation or fibrosis were categorized as the significant histological changes group, whereas those without such conditions were categorized as the non-significant histological changes group.

### Antiviral Treatment Regimens and Monitoring

Antiviral treatment regimens were as follows: (1) Nucleoside (acid) analogs (NAs): entecavir (ETV) 0.5 mg/day, taken orally on an empty stomach; tenofovir disoproxil fumarate (TDF), 300 mg/day, taken orally with meals; tenofovir alafenamide fumarate (TAF), 25 mg/day, taken orally with meals. (2) Pegylated interferon alpha-2a (Peg IFN-α-2a), 180 μg/week or 135 μg/week, subcutaneous injection, 48 weeks.

For other patients who did not require antiviral treatment, regular monitoring, including routine blood examinations, serum biochemical examination, HBV serological markers, HBV DNA levels, and liver stiffness measurements, were conducted every 3 months. Antiviral therapy was promptly initiated if the patient’s ALT levels were elevated.^2^

### Primary and Secondary Endpoints

The primary endpoint was virological response, defined as HBV DNA levels below 20 IU/mL. The secondary endpoints were as follows: (1) Virologic relapse, defined as HBV DNA levels rebounding to ≥ 500 IU/mL. (2) LLV, defined as detectable HBV DNA after 48 weeks of antiviral treatment, but < 2,000 IU/mL.^2^ (3) Hepatic events, including the development of liver cirrhosis and liver cancer. The follow-up period started from the initiation of antiviral treatment and ended in June 2024.

### Statistical Analysis

Statistical analysis was carried out utilizing SPSS software ver. 26.0 (IBM SPSS Corp.; Armonk, NY, USA). For continuous variables, the mean ± SD was used to describe those with a normal distribution, while the median (upper quartile, lower quartile) was applied for the non-normally distributed ones. Categorical variables were reported as counts and percentages (n, %). The independent samples *t*-test was used for continuous variables with a normal distribution, whereas the Mann–Whitney *U* test was utilized for non-normally distributed ones. For categorical variables, the chi-square test or Fisher’s exact test was implemented. Additionally, binary logistic regression analysis was conducted to identify predictors associated with significant liver inflammation or fibrosis. The receiver operating characteristic (ROC) curve was conducted to determine the area under the curve (AUC) and cut-off values. A *P* value of less than .05 was considered statistically significant.

## Results

### Baseline Characteristics of Study Group

Finally, a total of 70 patients with chronic HBV infection were enrolled. The median age was 40.5 (range: 31-60) years, and 38 patients (54.3%) were male. The results of blood routine, serum biochemical examinations were all within the normal reference ranges, the mean value of HBV DNA was 4.0 ± 0.9 log_10_IU/mL, and HBsAg was 2.8 ± 0.8 log_10_IU/mL. Regarding liver histopathological results, as shown in Table 1, 30 patients (42.9%) presented neither significant liver inflammation nor fibrosis, 1 patient (1.4%) had significant liver inflammation alone, 19 patients (27.1%) had significant liver fibrosis alone, and 20 patients (28.6%) showed both significant liver inflammation and fibrosis.

### Comparison of Pathological Changes and Other Clinical Data

The patients were classified into 2 groups according to their liver inflammation score (G): non-significant liver inflammation group (n = 49) and significant liver inflammation group (n = 21). As shown in Table 2, there were no significant differences in age, gender, white blood cells (WBC), hemoglobin (Hb), platelets (PLT), prothrombin time (PT), AFP, total bilirubin (TBil), ALT, γ-glutamyltransferase (GGT), alkaline phosphatase (ALP), HBV DNA levels, and HBsAg levels between the 2 groups (*P* > .05). However, compared with the non-significant liver inflammation group, the significant liver inflammation group had higher levels of aspartate aminotransferase (AST), but lower levels of red blood cells (RBC) (*P* < .05).

Based on the liver fibrosis score (S), the patients were divided into 2 groups: non-significant liver fibrosis (n = 31) and significant liver fibrosis group (n = 39). In terms of gender, WBC, RBC, Hb, PLT, PT, AFP, TBil, ALP, HBV DNA levels, and HBsAg levels, no significant differences were observed between the 2 groups (*P* > .05). As shown in Table 3, patients with significant liver fibrosis were found to be older than those without significant liver fibrosis (*P* < .05). Moreover, compared with the non-significant liver fibrosis group, the significant liver fibrosis group had higher levels of ALT, AST, and GGT (*P* < .05).

### Factors Related to Significant Liver Inflammation and Fibrosis

Subsequently, in order to identify the related factors of significant liver inflammation and significant liver fibrosis, multivariate binary logistic regression analyses were conducted. As shown in Table 4, the results demonstrated that RBC and AST were identified as independent predictors of significant liver inflammation. Specifically, RBC was a protective factor (OR(95%CI): 0.34(0.13-0.90), *P* = .03), while AST was a risk factor (OR(95%CI): 1.19(1.05-1.34), *P* = .006). Additionally, AST was also a risk factor for significant liver fibrosis (OR(95%CI): 1.24(1.06-1.47), *P* = .01).

**Figure 2.**
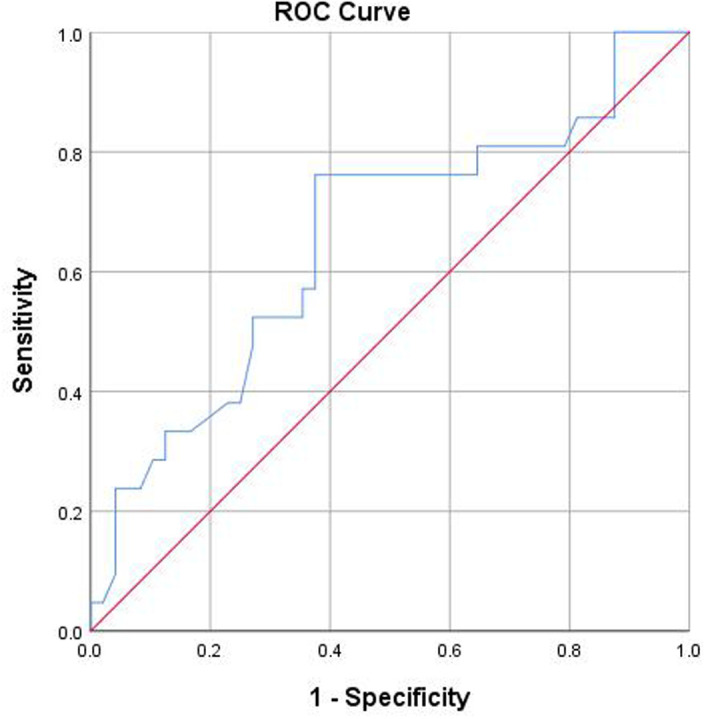
The prediction of significant liver inflammation was analyzed using an ROC curve with RBC. The AUC value was 0.65. The cut-off value was 4.70×10^12/L. Abbreviations: AUC, area under the receiver operating characteristic curve; RBC, red blood cells; ROC, receiver operating characteristic.

**Figure 3.**
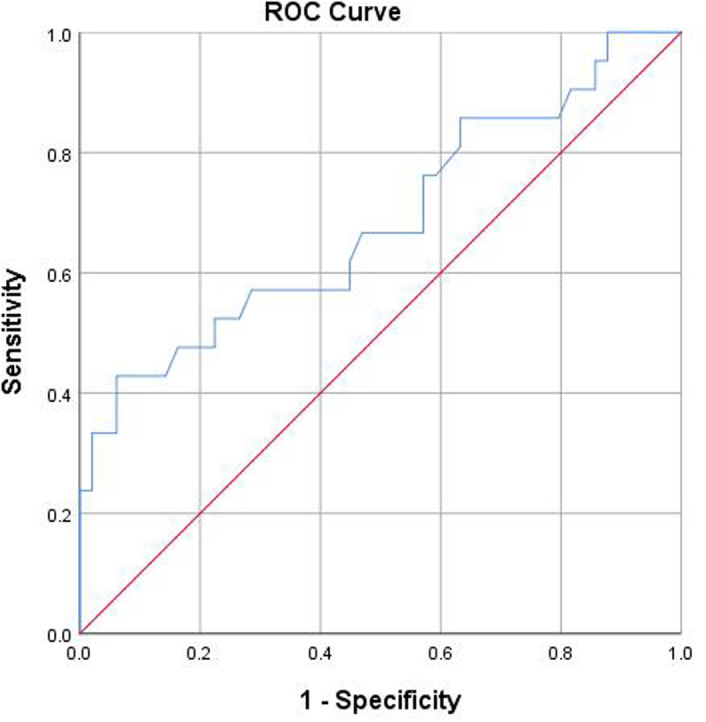
The prediction of significant liver inflammation was analyzed using an ROC curve with AST. The AUC value was 0.68. The cut-off value was 25.85 U/L. Abbreviations: AST, aspartate aminotransferase; AUC, area under the receiver operating characteristic curve; ROC, receiver operating characteristic.

**Figure 4.**
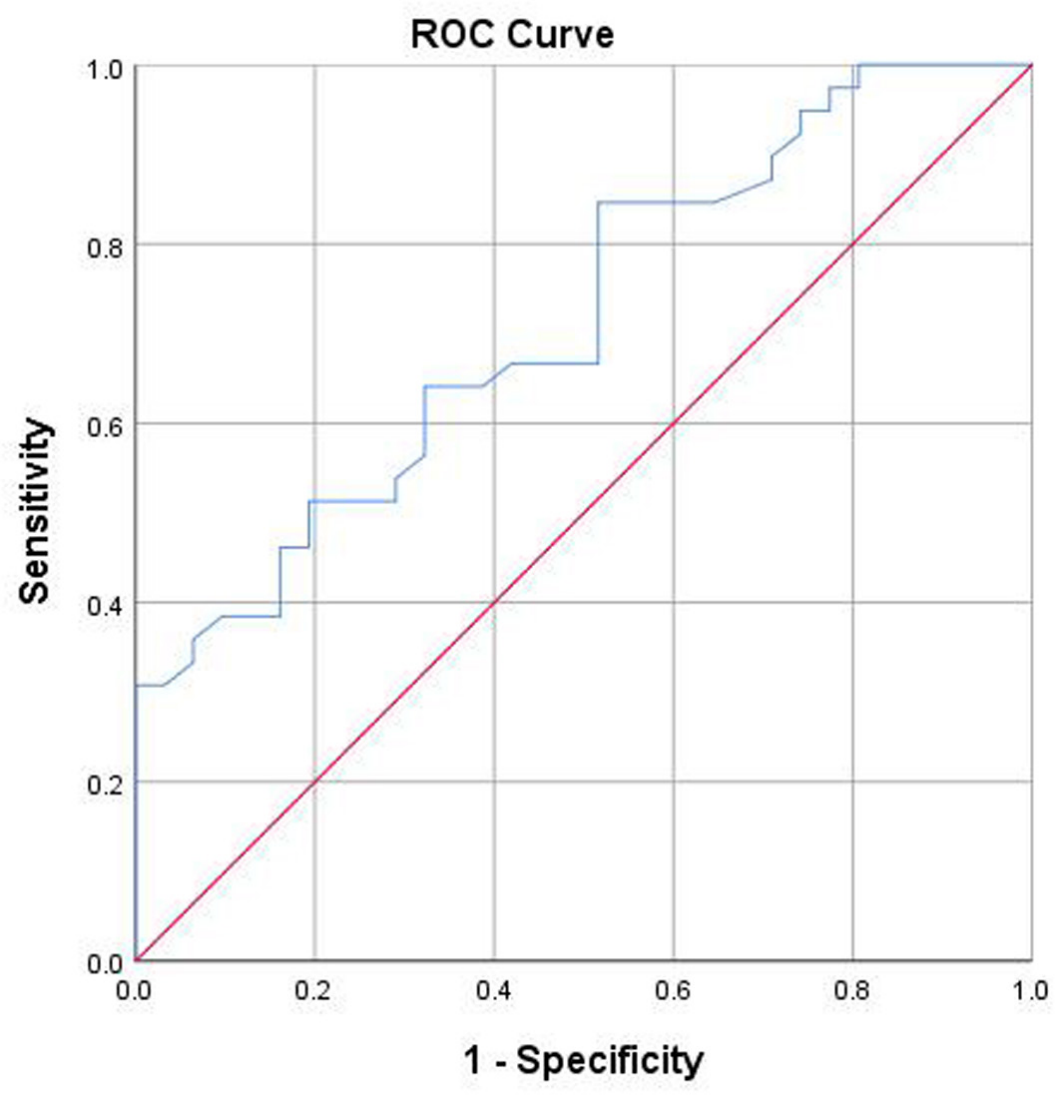
The prediction of significant liver fibrosis was analyzed using an ROC curve with AST. The AUC value was 0.75. The cut-off value was 19.15 U/L. Abbreviations: AST, aspartate aminotransferase; AUC, area under the receiver operating characteristic curve; ROC, receiver operating characteristic.

As illustrated in Figures 2, 3, and 4, ROC curve analyses were performed to assess diagnostic performance. The AUC of significant liver inflammation diagnosed by RBC and AST were 0.65 (95%CI: 0.51-0.80) and 0.68 (95%CI: 0.54-0.83), while the corresponding cut-off values were 4.70×10^12^/L and 25.85 U/L. In addition, AST had an AUC value of 0.72 (95%CI: 0.60-0.83) for diagnosing significant liver fibrosis, with a cut-off value of 19.15 U/L.

### Clinical Characteristics and Antiviral Efficacy of Patients

Among the 70 patients, 62 patients (88.57%) received antiviral treatment after liver biopsy, with a median follow-up time of 91.6 weeks (range: 40-254 weeks). Regarding the antiviral treatment regimen, 45 patients (72.6%) were treated with NAs alone, while 17 patients (27.4%) were treated with a combination of Peg IFN-α-2a and NAs. In terms of liver histology before antiviral treatment, 1 patient (1.6%) had only significant inflammation, 19 patients (30.6%) had only significant fibrosis, 20 patients (32.3%) had both significant inflammation and fibrosis, and 22 patients (35.5%) had neither. In addition, the 8 patients without antiviral treatment all exhibited non-significant inflammation and fibrosis.

After 24 weeks of antiviral treatment, the HBV DNA negative conversion rates were all above 80%. Based on the liver inflammation score (G) and liver fibrosis score (S), patients were divided into groups with non-significant and significant histological changes, accounting for 35.5% and 64.5%. As shown in Table 5, no statistically significant differences were found between the 2 groups regarding age, gender, HBV DNA levels, HBsAg levels, antiviral treatment regimen, HBV DNA negative conversion rate, HBV DNA negative conversion time, and LLV at 48 weeks (*P* > .05).

### The Occurrence of Adverse Events

In the group of non-significant histological changes, 2 patients had LLV and their HBV DNA remained detectable throughout the follow-up period. It is hypothesized that their high pre-treatment HBV DNA levels (9.78×10^7^ IU/mL and 6.54 × 10^8^ IU/mL, respectively) served as the underlying contributing factors for the persistence of LLV. Similarly, 2 patients with significant histological changes developed LLV at 48 weeks during the antiviral treatment. Notably, one of them achieved HBV DNA negative conversion at 72 weeks.

In addition, 2 patients with significant histological changes developed HBV DNA relapse at 96 weeks of antiviral treatment, and drug resistance sites (rtV173L, rtL180M, and rtM204V/I) were detected. After adjusting antiviral drugs, their HBV DNA turned negative at 120 weeks. Notably, during the follow-up period, none of the 62 patients developed liver cancer. Moreover, no serious complications, such as renal injury or osteoporosis, resulted in drug withdrawal.

Among the 8 patients without antiviral treatment, 3 patients showed elevated ALT 1-2 years after liver biopsy. However, their HBV DNA turned negative within 24 weeks after initiating antiviral treatment. The other 2 patients maintained normal ALT throughout the follow-up period, and the remaining 3 patients were lost to follow-up.

## Discussion

The results showed that among HBeAg-negative CHB patients aged over 30 in the indeterminate phase, over half had significant liver inflammation or fibrosis, consistent with most studies which reported that approximately 50% of patients in the indeterminate phase had significant liver inflammation or fibrosis.^5^^-^^8^ Notably, patients with significant liver fibrosis were older than those without significant liver fibrosis, consistent with the meta-analysis by Lai et al,^13^ suggesting that liver damage worsens with age. Therefore, it is necessary for patients with chronic HBV infection over 30 years old to initiate antiviral treatment as soon as possible.

This study found that RBC was related to significant liver inflammation. Since there were no differences in age and gender between the groups with and without significant liver inflammation, the influence of gender or age on the differences in RBC can be excluded. Similarly, Mahdavi et al^14^ and Xie et al^15^ reported that RBC is related to the degree of liver damage, which may contribute to improved liver function. Inflammation influences erythropoiesis by several mechanisms, such as disruption of bone marrow function of erythrocyte differentiation and maturation, suppression of erythrocyte maturation by inflammatory cytokines, reduction of erythrocytes caused by hypersplenism in some patients with cirrhosis, acceleration of erythrocyte apoptosis, decrease of erythropoietin production, and inhibition of iron absorption.^16^^-^^20^

This study found that AST was associated with significant liver inflammation and fibrosis. AST demonstrated a notable ability to predict significant liver inflammation and fibrosis, with a relatively stronger predictive power for fibrosis. This may be because AST, which is predominantly located in hepatocyte mitochondria, is a more sensitive indicator for assessing the severity and duration of hepatocyte damage compared to ALT.^21^ In addition, Feng et al^22^ reported that AST was identified as an independent predictor for significant liver injury, and Zhang et al^23^ reported that AST/ALT levels are correlated with the severity of liver inflammation and fibrosis and poor prognosis. Currently, the WHO 2024 guidelines for chronic hepatitis B infection management and Chinese 2022 guidelines for chronic hepatitis B prevention and treatment both recommend lowering transaminase levels as a strategy to expand the scope of antiviral treatment.^2^^,^^24^

Moreover, considering the limitations of relying solely on ALT levels, several studies have shown that more than 30% of patients with normal ALT have significant liver inflammation or significant liver fibrosis.^6^^,^^25^^,^^26^ This suggests that using abnormal ALT alone as an indication to initiate antiviral therapy is not feasible. In this study, it was found that AST was associated with significant liver inflammation and significant liver fibrosis among CHB patients in the indeterminate phase with normal ALT. This finding highlights the potential value of AST when ALT levels are within the normal range. Perhaps in patients with normal ALT, for diagnosing significant histological changes, the diagnostic efficacy of AST is superior to ALT. Furthermore, AST and ALT can be combined for better assessment. For example, the non-invasive indicator FIB-4, which incorporates AST along with ALT, platelet count, and age, is used for evaluating liver fibrosis.^27^

HBV DNA is an important indicator for follow-up monitoring of patients with chronic hepatitis B, reflecting HBV replication and infectivity.^2^ Li et al^28^ and Jiang et al^29^ reported that HBV DNA is correlated with liver pathology, whereas Xing et al^30^ and Leowattana et al^31^ reported that the correlation is weak or not. The reason for this discrepancy is not clear, and most studies included patients with chronic hepatitis B in the determinate phases. However, patients in this study were HBeAg-negative CHB patients in the indeterminate phase and it was found that the HBV DNA was not correlated with significant liver inflammation or fibrosis. In addition, Tseng et al^32^reported that HBV DNA of patients in the indeterminate phase is not correlated with the occurrence of HCC after Cox regression analysis, which indicates that HBV DNA cannot reflect the severity of liver damage among patients in the indeterminate phase, let alone serve as an indicator to initiate or discontinue antiviral treatment.

Among the 62 patients who received antiviral treatment, the negative conversion rate of HBV DNA reached 80% or higher regardless of significant histological changes, which was consistent with the high negative conversion rate for CHB patients with negative HBeAg and normal ALT reported by Li et al^33^and Wei et al.^34^ Regarding the incidence of adverse events, no significant difference was observed in the rate of LLV between the 2 groups. This suggested that the occurrence of LLV was not associated with the presence or absence of significant histological changes. This conclusion was based on the fact that the baseline characteristics and treatment conditions of the 2 groups were comparable, and no other confounding factors were found to influence the LLV proportion. Kim et al^35^ reported that patients with normal ALT are more likely to develop LLV during antiviral treatment, and LLV is an independent risk factor for HCC. And Sun et al^36^ reported that persistent LLV promotes fibrosis progression during therapy. However, due to the lack of post-treatment liver biopsies in this cohort, a detailed longitudinal analysis of changes in hepatic fibrosis and inflammation during antiviral therapy could not be conducted. Considering these findings and limitations, and given the potential risks of liver damage even in CHB patients in the indeterminate phase with normal ALT, it is advisable to initiate antiviral treatment promptly. During the antiviral treatment, long-term monitoring and regular assessment of HBV DNA are required, which can promptly detect the occurrence of LLV, drug resistance, and other adverse events. According to the 2022 Chinese guideline, it is recommended to adjust the treatment regimen or drug combination if LLV occurs.^2^

The major strength of this study is that the study population are CHB patients in the indeterminate phase with normal ALT and negative HBeAg. However, the studies focusing on such patients are limited, and there remains controversy regarding the administration of antiviral treatment for them. Notably, this study found that more than 30% of them have significant liver inflammation or fibrosis, and antiviral treatment is effective for them. Nonetheless, this study has some limitations. First, the small sample size and the data of only a single center lead to statistical bias, so the sample size needs to be expanded to enhance the representativeness of data and reduce the potential statistical bias. Second, this study is a retrospective study, so the real-time records of the events may be biased.

These findings suggest that RBC and AST may be associated with significant liver inflammation, and AST may also be associated with significant liver fibrosis. Further consideration can be given to reducing the Upper limit of normal (ULN) of ALT and AST to better manage these patients. In addition, antiviral treatment is effective in suppressing HBV replication for HBeAg-negative indeterminate phase chronic HBV infection patients aged over 30 years, but attention should be paid to the occurrence of LLV and drug resistance.

## Figures and Tables

**Table 1.** Baseline Characteristics of the Study Group

Variables	Total (n = 70)
Age [*M* (*P*_25_,*P*_75_)], years	40.5 (35.7,48.2)
Gender, male, n (%)	38 (54.3)
WBC (*X̅* ± *s*), ×10^9^/L	5.6 ± 1.6
RBC (*X̅* ± *s*), ×10^12^/L	4.7 ± 0.6
Hb [*M* (*P*_25_,*P*_75_)], g/L	140 (125,155)
PLT (*X̅* ± *s*), 10^9^/L	192.0 ± 57.4
PT [*M* (*P*_25_,*P*_75_)], seconds	11.6 (10.8,12.2)
AFP [*M* (*P*_25_,*P*_75_)], ng/mL	2.6 (1.7,7.6)
TBil [*M* (*P*_25_,*P*_75_)], μmol/L	13.8 (10.3,18.6)
ALT (X̅ ± *s*), U/L	23.4 ± 8.4
AST [*M* (*P*_25_,*P*_75_)], U/L	21.1 (18.7,23.9)
GGT [*M* (*P*_25_,*P*_75_)], U/L	20.5 (13.9,29.7)
ALP [*M* (*P*_25_,*P*_75_)], U/L	60.0 (49.8,78.3)
HBV DNA (*X̅* ± *s*), log_10_IU/mL	4.0 ± 0.9
HBsAg (*X̅* ± *s*), log_10_IU/mL	2.8 ± 0.8
Pathological changes	
Non-significant liver inflammation and fibrosis, n (%)	30 (42.9)
Only significant liver inflammation, n (%)	1 (1.4)
Only significant liver fibrosis, n (%)	19 (27.1)
Significant liver inflammation and fibrosis, n (%)	20 (28.6)

*M* (P25, P75), median (upper quartile, lower quartile); (*X̅ ± s*), mean ± SD; AFP, alpha fetoprotein; ALP, alkaline phosphatase; ALT, alanine aminotransferase; AST, aspartate aminotransferase; GGT, glutamyltransferase; Hb, hemoglobin; PLT, platelets; PT, prothrombin time; RBC, red blood cells; TBil, total bilirubin; WBC, white blood cells.

**Table 2.** Comparison of Baseline Characteristics of patients with Non-significant and Significant Liver Inflammation

Variables	Non-significant Liver Inflammation (n = 49)	Significant Liver Inflammation (n = 21)	*t*/*χ*^2^/*Z*	*P*
Age [*M* (*P*_25_,*P*_75_)], years	40.0 (35.0,47.0)	45 (37.5,51)	1.34	.18
Gender, male, n (%)	28 (57.1)	10 (47.6)	0.54	.46
WBC [*M* (*P*_25_,*P*_75_)], ×10^9^/L	5.8 (4.3,6.6)	5.9 (4.1,6.9)	−0.20	.84
RBC (*X̅* ± *s*), ×10^12^/L	4.8 ± 0.5	4.4 ± 0.7	2.39	.02
Hb [*M* (*P*_25_,*P*_75_)], g/L	149.5 (127.3,156.8)	137.0 (118.0,148.0)	−1.94	.053
PLT [*M* (*P*_25_,*P*_75_)], ×10^9^/L	191.5 (154.0,239.0)	178.0 (127.0,229.5)	−1.45	.15
PT [*M* (*P*_25_,*P*_75_)], seconds	11.5 (10.8,12.0)	11.9 (10.8,13.1)	1.03	.30
AFP [*M* (*P*_25_,*P*_75_)], ng/mL	2.5 (1.8,5.3)	3.8 (1.5,12.7)	1.18	.24
TBil [*M* (*P*_25_,*P*_75_)], μmol/L	13.5 (10.1,18.4)	14.6 (11.2,18.7)	1.19	.24
ALT (*X̅* ± *s*), U/L	22.9 ± 8.1	24.5 ± 9.4	−0.72	.48
AST (X̅ ± *s*), U/L	20.5 ± 3.8	25.8 ± 8.3	−2.78	.01
GGT [*M* (*P*_25_,*P*_75_)], U/L	20.5 (13.2,29.0)	20.0 (16.2,48.6)	0.85	.39
ALP [*M* (*P*_25_,*P*_75_)], U/L	60.0 (48.0,74.0)	68.4 (55.8,82.0)	1.01	.31
HBV DNA [*M* (*P*_25_,*P*_75_)], log_10_IU/mL	3.8 (3.4,4.3)	3.7 (3.0,5.1)	0.36	.72
HBsAg [*M* (*P*_25_,*P*_75_)], log_10_IU/mL	2.6 (2.2,3.3)	2.8 (2.2,3.3)	0.24	.81

*M* (P25, P75), median (upper quartile, lower quartile); (*X̅ ± s*), mean ± SD;AFP, alpha fetoprotein; ALP, alkaline phosphatase; ALT, alanine aminotransferase; AST, aspartate aminotransferase; GGT, glutamyltransferase; Hb, hemoglobin; PLT, platelets; PT, prothrombin time; RBC, red blood cells; TBil, total bilirubin; WBC, white blood cells.

**Table 3.** Comparison of Baseline Characteristics of Non-significant and Significant Liver Fibrosis

Variables	Non-significant Liver Fibrosis (n = 31)	Significant Liver Fibrosis (n = 39)	*t*/*χ*^2^/*Z*	*P*
Age (*X̅* ± *s*), years	40.1 ± 6.5	43.8 ± 7.6	−2.13	.04
Gender, male, n (%)	16 (51.6)	22 (56.4)	0.16	.69
WBC [*M* (*P*_25_,*P*_75_)], ×10^9^/L	5.9 (4.0,6.3)	5.6 (4.5,6.9)	0.68	.49
RBC (*X̅* ± *s*), ×10^12^/L	4.7 ± 0.5	4.6 ± 0.7	0.22	.83
Hb [*M* (*P*_25_,*P*_75_)], g/L	140.5 (123.8,155.0)	140.0 (128.0,155.0)	0.39	.70
PLT (*X̅* ± *s*), ×10^9^/L	204.6 ± 65.2	182.2 ± 49.3	1.62	.11
PT [*M* (*P*_25_,*P*_75_)], seconds	11.7 (10.9,12.1)	11.4 (10.8,12.5)	−0.59	.56
AFP [*M* (*P*_25_,*P*_75_)], ng/mL	2.5 (1.7,3.8)	3.3 (1.9,11.8)	1.50	.13
TBil [*M* (*P*_25_,*P*_75_)], μmol/L	11.2 (10.1,17.9)	14.1 (11.1,18.8)	1.00	.32
ALT [*M* (*P*_25_,*P*_75_)], U/L	20.4 (13.4,27.9)	25.2 (17.1,34.9)	2.12	.03
AST [*M* (*P*_25_,*P*_75_)], U/L	20.3 (16.9,22.2)	23.0 (19.4,27.4)	3.09	.002
GGT [*M* (*P*_25_,*P*_75_)], U/L	18.0 (13.0,25.6)	21.7 (17.4,39.8)	1.99	.046
ALP [*M* (*P*_25_,*P*_75_)], U/L	58.0 (46.0,73.0)	61.0 (55.0,82.0)	1.30	.20
HBV DNA [*M* (*P*_25_,*P*_75_)], log_10_IU/mL	3.8 (3.5,4.2)	4.0 (3.2,4.8)	0.14	.89
HBsAg [*M* ( *P*_25_,*P*_75_)], log_10_IU/mL	2.6 (2.1,3.4)	2.8 (2.3,3.3)	0.41	.68

*M* (P25, P75), median (upper quartile, lower quartile); (*X̅ ± s*), mean ± SD;AFP, alpha fetoprotein; ALP, alkaline phosphatase; ALT, alanine aminotransferase; AST, aspartate aminotransferase; GGT, glutamyltransferase; Hb, hemoglobin; PLT, platelets; PT, prothrombin time; RBC, red blood cells; TBil, total bilirubin; WBC, white blood cells.

**Table 4.** Multivariate Logistic Regression Analysis of Significant Liver Inflammation and Fibrosis

Variables	Significant Liver Inflammation		Significant Liver Fibrosis	
	OR (95% CI)	*P*	OR (95% CI)	*P*
Age, years			1.07 (0.99,1.16)	.08
RBC, ×10^12^/L	0.34 (0.13,0.90)	.03		
ALT, U/L			1.02 (0.93,1.12)	.65
AST, U/L	1.19 (1.05,1.34)	.006	1.24 (1.06,1.47)	.01
GGT, U/L			1.04 (0.99,1.09)	.10

ALT, alanine aminotransferase; AST, aspartate aminotransferase; GGT, glutamyltransferase; RBC, red blood cells; OR, odd ratio; CI, confidence interval.

**Table 5.** Basic Characteristics and Antiviral Treatment Condition of patients with Non-significant Histological Changes Versus Significant Histological Changes

Variables	Non-significant histological changes (n = 22)	Significant histological changes (n = 40)	*t*/*χ*^2^/*Z*	*P*
Age (X̅ ± *s),* years	41.2 ± 6.4	43.7 ± 7.6	−1.30	.20
Gender, male, n (%)	11 (50.0)	23 (57.5)	0.32	.57
HBV DNA [*M* (*P*_25_,*P*_75_)], log_10_IU/mL	3.8 (3.5,4.3)	3.9 (3.2,4.8)	−0.07	.95
HBsAg [*M* (*P*_25_,*P*_75_)], log_10_IU/mL	2.5 (1.9,3.1)	2.8 (2.3,3.3)	1.40	.16
Antiviral treatment regimens			1.37	.24
NAs, n (%)	14 (63.6)	31 (77.5)		
NAs + Peg IFN-α-2a, n (%)	8 (36.4)	9 (22.5)		
Virological response				
HBV DNA turned negative within				
24 weeks, % [n (total cases)]	81.8 (18/22)	85.0 (34/40)	0.11	1.00
48 weeks, %[n, (total cases)]	90.9 (20/22)	95.0 (38/40)	0.39	.61
72 weeks (n = 47), % [n (total cases)]	88.2 (15/17)	96.7 (29/30)	1.29	.54
96 weeks (n = 42), % [n (total cases)]	87.5 (14/16)	88.5 (23/26)	0.96	.55
144 weeks (n = 28), %[n (total cases)]	80.0 (8/10)	94.4 (17/18)	1.40	.53
HBV DNA negative conversion time [*M* (*P*_25_,*P*_75_)], weeks	11.0 (5.6,22.9)	17.9 (6.1,24.0)	0.52	.60
LLV at 48 weeks, n (%)	2 (9.1)	2 (5.0)	0.39	.61

*M* (P25, P75), median (upper quartile, lower quartile); (*X̅ ± s*), mean ± SD; LLV, low-level viremia; NAs, nucleoside (acid) analogs; Peg IFN-α-2a, pegylated interferon alpha-2a.

## Data Availability

The data that support the findings of this study are available on request from the corresponding author.
